# Editorial for the Special Issue on Micro and Nanosensors: Fabrication, Applications and Performance Enhancements

**DOI:** 10.3390/mi15091124

**Published:** 2024-09-02

**Authors:** Joanna Smajdor

**Affiliations:** Faculty of Materials Science and Ceramics, AGH University of Krakow, al. Mickiewicza 30, 30-059 Cracow, Poland; smajdorj@agh.edu.pl

The advancement of electrochemical sensors is an expanding area of research that is drawing growing interest from scientists worldwide. Recently, there has been a notable focus on making these sensors smaller without compromising their sensitivity and selectivity. Many new designs incorporate innovative materials as sensing components, offering additional benefits like flexibility and diverse shapes for these sensors [1,2,3]. Micro and nanosensors are used for highly sensitive measurements of chemical, physical, or biological quantities, while maintaining small sensor sizes, on the micro and nanoscale, respectively. The most typical parameters, but not the only ones, that can be measured using such kinds of sensors are, for example, pressure [4,5,6], humidity [7,8,9], concentration [10,11,12], or temperature [13,14,15], which makes them have wide applications in various fields, including electronics, medicine, automotive, and industry. Given the multitude of parameters that can be determined using micro and nanosensors, they are ubiquitous in our lives, even though we cannot always be aware of them.

This Special Issue consists of eight papers, focused on the topic of preparation, application, and performance of micro and nanosensors, such as laser-inscribed graphene electrodes [16], glucose sensors [17], tunnel magnetoresistance current sensors [18], quartz resonant pressure sensors [19], MEMS fluxgate sensors [20], metamaterial structures [21], micro 4D imaging sensors [22], and the electrochemical usage of microscale sensors for the determination of antidiabetic drugs [23].

The first study by Tang et al. [16] investigates batch-to-batch variation in laser-inscribed graphene (LIG) electrodes, which are used for electrochemical sensing in microelectronic applications like supercapacitors, sensors, and triboelectric generators. LIG electrodes were fabricated using a CO_2_ laser on polyimide film and characterized through methods such as goniometry, stereomicroscopy, and cyclic voltammetry. The results showed that batch-to-batch variation in LIG electrodes was less than 5% in terms of hydrophobicity and electrochemical properties when using standard electrodes. The metallization of LIG electrodes also improves peak current and capacitance, increasing variability to about 30%. Another paper by Yang et al. [17] proposes a sensor using a complementary split ring resonator (CSRR) for non-destructive blood glucose testing. The CSRR structure enhances the electromagnetic field between the split rings, improving the sensor’s performance. Also, a 3D-printed holder was used to test glucose concentrations. Experimental results show a clear linear relationship between glucose concentration and insertion loss at the resonant frequency, confirming the feasibility of this CSRR-based sensor for non-invasive glucose detection. A study by Wang et al. [18] introduces a high-resolution modulation–demodulation test method to improve the accuracy of weak current detection in tunnel magnetoresistance current sensors by mitigating low-frequency noise and interference from ambient magnetic fields. The technique shifts low-frequency noise to the high-frequency range, enabling more effective filtering and isolating the target signal. A Simulink model was developed for the method, and the results of practical tests show that the method effectively reduces noise impact on TMR sensors and could be applied to other resistive devices. The paper by Yao et al. [19] focuses on improving the accuracy of pressure measurements using AT-cut quartz, a highly precise and durable pressure transducer, by addressing its temperature sensitivity. To compensate for temperature effects, three machine learning algorithms—multivariate polynomial regression (MPR), multilayer perceptron networks, and support vector regression—were applied to establish predictive models. Experimental validation showed that all methods effectively improved accuracy, with the MPR model performing the best. Additionally, an automatic compensation system was developed for real-time processing and calibration, enhancing the industrial scalability of resonant pressure sensors. This approach offers a promising solution for temperature compensation in such sensors. A study by Yang et al. [20] presents a MEMS fluxgate sensor that utilizes liquid casting to form a 3D solenoid coil with enhanced line width and thickness, improving sensor performance compared to traditional electroplating methods. The sensor is based on a closed-loop Fe-based amorphous alloy core. By designing the sensor structure according to liquid casting parameters and using MagNet simulations, optimal excitation conditions and sensitivity were determined. The fabricated sensor demonstrated high sensitivity, low noise, wide bandwidth, and low power consumption, making it more efficient than similar MEMS fluxgate sensors in size and performance. This paper by Zou et al. [21] introduces a novel tuning strategy using a pneumatically actuated metamaterial to enable on-demand polarization manipulation at terahertz frequencies. By adjusting the actuation pressure, the device can switch between three types of polarization conversion capabilities. These capabilities include converting between linear and circular polarization (acting as a quarter-wave plate), inverting the handedness of circular polarization (as a helicity inverter), and maintaining the handedness (as a helicity keeper) between incidence and reflection. The paper also explores the intrinsic tuning mechanism behind this polarization control. Another paper by Jiang et al. [22] presents a novel approach to spectral and depth (SAD) imaging using a snapshot narrow band imaging (SNBI) method, addressing the challenge of achieving accurate depth estimation and spectral imaging from a single image without increasing sensor size. The study introduces a micro 4D imaging (M4DI) sensor, combining a monochromatic imaging sensor with a narrow-band microarray spectral filter. This sensor, which retains the appearance and size of traditional sensors, captures wavelength-dependent spectral aberration for simultaneous spectral and depth imaging. A simple remapping algorithm separates the image into four spectral bands, and a depth estimation algorithm generates 3D data with a dense depth map in every exposure. The M4DI sensor outperforms existing SAD methods by offering easy implementation, low computational burden, and cost efficiency. The last article by Fendrych et al. [23] reviews the current knowledge on electrochemical methods for detecting active substances in drugs used to treat type 1 and type 2 diabetes. These methods offer a sensitive and cost-effective alternative to traditional, more expensive analytical techniques. The detection relies on oxidation or reduction processes at the surface of working electrodes, which are often enhanced with modifications like nanoparticles or conducting polymers to improve sensitivity. These electrochemical assays are capable of analyzing antidiabetic compounds in diverse samples, including pharmaceutical products and human bodily fluids, making them highly useful in clinical and pharmaceutical settings.

I would like to take this opportunity to thank all the authors who have submitted their papers to this Special Issue and to thank all the reviewers for their contributions in evaluating the submitted papers and for their efforts in improving the quality of the manuscripts. Also, I am very grateful to the *Micromachines* Editorial Board for giving me the opportunity to be a guest editor in this Special Issue.
